# Digital health and health equity: How digital health can address healthcare disparities and improve access to quality care in Africa

**DOI:** 10.34172/hpp.42822

**Published:** 2024-03-14

**Authors:** Ibraheem Olasunkanmi Qoseem, Olalekan John Okesanya, Noah Olabode Olaleke, Bonaventure Michael Ukoaka, Blessing Olawunmi Amisu, Jerico Bautista Ogaya, Don Eliseo Lucero-Prisno III

**Affiliations:** ^1^Department of Medical Laboratory Science, General Hospital, Ilesha, Osun State, Nigeria; ^2^Department of Public Health and Maritime Transport, University of Thessaly, Volos, Greece; ^3^Department of Medical Laboratory Science, Obafemi Awolowo University Teaching Hospitals Complex, Ile Ife, Osun State, Nigeria; ^4^Department of Internal Medicine, Asokoro District Hospital, Abuja, Nigeria; ^5^Department of Medical Laboratory Science, Uniosun Teaching Hospital, Osogbo, Osun State; ^6^Department of Medical Technology, Far Eastern University, Manila, Philippines; ^7^Department of Global Health and Development, London School of Hygiene and Tropical Medicine, London, United Kingdom

**Keywords:** Health equity, Digital health, Health disparities, Digital innovations, Artificial intelligence (AI), Healthcare delivery

## Abstract

The healthcare industry is constantly evolving to bridge the inequality gap and provide precision care to its diverse population. One of these approaches is the integration of digital health tools into healthcare delivery. Significant milestones such as reduced maternal mortality, rising and rapidly proliferating health tech start-ups, and the use of drones and smart devices for remote health service delivery, among others, have been reported. However, limited access to family planning, migration of health professionals, climate change, gender inequity, increased urbanization, and poor integration of private health firms into healthcare delivery rubrics continue to impair the attainment of universal health coverage and health equity. Health policy development for an integrated health system without stigma, addressing inequalities of all forms, should be implemented. Telehealth promotion, increased access to infrastructure, international collaborations, and investment in health interventions should be continuously advocated to upscale the current health landscape and achieve health equity.

## Introduction

 By standard, healthcare is a basic human right, but not everyone has equal access to it. The unfair or unequal access to healthcare between populations shows the ways in which socioeconomic power distribution determines individual risks and opportunities, including access to stable and safe housing, food, and water, and access to appropriate health services.^1^ Inequalities in access to healthcare, usage, and results contribute significantly to global chronic disease and death burden, alcohol and drug abuse, and mental and psychological well-being and have far-reaching consequences for the standard of life, livelihood, schooling, and wellness, especially among cultural and racial monitories, women, the LGBTQ (Lesbian, Gay, Bisexual, Transgender, and Queer) population, and native civilians in Africa.^2,3^ Significant health disparities have been reported globally between low- and middle-income countries (LMICs), especially among populations at risk across the continent. Inequalities regarding healthcare research and clinical application lead to mistrust and alienation, which further restrict access irrespective of whether facilities are technically available.^4^ Health equity refers to the significant efforts channelled towards eliminating health disparities. The concept of health equity is viewed as a human right and an effort to fight against structural violence and institutional discrimination.^5^

 The fast-rising digital revolution has yielded greater transformations across all sectors, including health. Its ripple effect has led to effortless communication and connectivity for millions of people. Notwithstanding, it is also a game changer in health as it has helped in bridging the wider gap in health delivery.^6,7^ Digital innovations in health include virtual health, mobile health apps (m-Health), wearable devices, the internet of medical things (IoMT), artificial intelligence (AI), machine learning (ML), and blockchain. With the promising breakthrough offered by digital health, addressing health disparities and improving healthcare access in Africa, especially in some rural communities, is practically feasible.^8,9^ In 2005, the World Health Organization (WHO) recognized the significance of information and communication technology (ICT) in the field of health. It issued an actionable plan for 2020-2025 to guarantee that electronic health care is established and executed in an approach that promotes equality, cost-effectiveness, and accessibility. But this has been far from reaching Africa.^10,11^ Hence, the study aims to critically assess the progress and challenges to achieving health equality in Africa and the roles of digital health technologies in bridging this gap, which aligns with global health goals to promote fairness and increase healthcare accessibility through innovative technological advances.

## Progress in achieving health equity in Africa

 Africa’s improvements in healthcare are gaining momentum, with a 50% rise in average life expectancy ranging from 41 to 60 years between 1960 and 2021, although it continues to lag below LMICs and OECD standards, showing optimism.^12^ Maternal mortality has decreased, but difficulties persist. Fertility rates have decreased, but access to family planning has been limited.^13^ Healthcare infrastructure, personnel resources, and funding increase but are not sufficient. Some countries implement healthcare reforms, such as insurance programmes, to decrease out-of-pocket expenditures and ensure more sustainable financing. The private sector is increasingly important in health finance, accounting for around half of all spending in Sub-Saharan Africa.^14,15^ Some countries are attempting to provide universal healthcare coverage in order to mitigate financial risks and enhance overall health results. Through the implementation of digital healthcare technologies in countries such as Kenya, South Africa, Egypt, and Nigeria, Africa is making strides towards health fairness. Helium Health, for example, has pioneered full health technology services such as diagnostics, electronic medical records, and telemedicine.^16-18^ These programmes influence over 1 million users and have delivered over 3 million USD in healthcare-related credit. During the Ebola epidemic, contact tracing technology has been critical in handling COVID-19 patients.^19^

 Significant technical developments have occurred in Africa, particularly in digital health solutions. Despite hurdles, the region will have received over US$ 4.8 billion in startup capital by 2022. This expansion reflects Africa’s untapped potential as well as technological opportunities to handle complicated concerns.^20^ According to projections, the African economy might grow by an additional US$ 1.4 trillion, outpacing growth rates in the United States and Europe. Cities such as Mauritius, Nairobi, and Kigali are at the forefront of this shift, especially in healthcare, where innovations such as drone delivery and mobile services are improving public health access. The confluence of health and financial services, such as MPESA in Kenya, is democratizing access to healthcare. The future of healthcare delivery in Africa is predicted to be shaped by smart cities in Lagos, Cape Town, and Nairobi. Africa’s digital employment is quickly expanding, with over 716 000 developers expected by 2021, with 38% working for multinational corporations.^20,21^ However, as elite talent migrates elsewhere for more lucrative possibilities, Africa faces obstacles. With over half of the world’s youth anticipated to be African by 2030, the potential for technological innovation is enormous. Significant investments in tech startups have resulted from the continent’s innovation explosion, which socioeconomic difficulties and possibilities have spurred.^22^ Despite the talent migration, growing African representation in the global digital giant provides the potential for incorporating the African context into product creation. Diasporan talent has the potential to contribute to the continent’s long-term growth. These developments emphasize the significance of widespread technological adoption and infrastructure development.^23^ Africa’s progress toward health equity is being propelled by a surge in innovation as a result of its particular socioeconomic situation. Despite obstacles, Africa has emerged as a technology solutions hub, with tech companies rapidly expanding and drawing investments totaling more than US$ 5.5 billion between 2021 and 2022, making Africa a prime site for revolutionary technologies.^24^

## Challenges to achieving health equity in Africa

 Africa faces significant problems in building sustainable and equitable healthcare systems. The continent’s inadequate health systems and institutions make it difficult to respond quickly to emergent issues. The high turnover of senior health authorities, insufficient resources, bad administration, and poor implementation all limit growth.^24,25^ The private sector makes substantial contributions to healthcare delivery, yet there is a significant gap in discussion and information sharing between the commercial and public sectors. Outdated regulatory frameworks and a lack of resources exacerbate enforcement challenges.^26,27^ Private healthcare facility inspections are disregarded, with only 20% establishing medical education criteria for individuals working in private hospitals. Rapid urbanization and an urban bias create an urgent demand for enlarged urban hospitals, putting further strain on already overburdened health-care systems. The continent is suffering from a severe shortage of competent health personnel, with the WHO estimating a 1.5 million gap in Africa.^27,28^ Significant obstacles are posed by the fall in development assistance, biased investment toward infectious diseases, and unproductive domestic public spending. Climate change, gender inequity, and increased urbanization worsen health inequities, disproportionately harming vulnerable populations.^29^ Inefficient resource allocation, inadequate governance, and a lack of evidence-based policies hinder improvement, necessitating a rethinking and rebuilding of health-care systems.^30^ Poor funding, poor institutional capacity, disparities in access to interventions, insufficient health data, and insufficient monitoring and evaluation capacity are on the rise. The region faces challenges in reducing child mortality and combating diseases such as HIV/AIDS and malaria.^16,25^ Access and quality of health care remain hampered by urban-rural and socioeconomic disparities. Inequalities in healthcare access, urban-rural divides, and a lack of crucial drugs all impede healthcare delivery.^1,31^

## Bridging health disparities: the role of digital health

 Digital technologies are rapidly proliferating and have witnessed wide application in the public health space in the last two decades.^32^ A diverse array of digital technologies has been implemented, targeting the transformation of public health services’ speed and cost-effectiveness. These technologies include smart wearables, social media, mobile apps, big data, and AI.^33^ The development of strategy frameworks by international and regional public health agencies to leverage the potential benefits of digital technologies to improve public health outcomes highlights the growing significance of digital technology in public health.^33^

 Digital health breakthroughs are quietly transforming well-being, particularly for neglected populations and socioeconomically disadvantaged groups who face the greatest need for health interventions.^34^ Conventional medical services, including scheduling appointments, seeking health consultations, virtual payment platforms, and digital medical reports generation, have all been included in mobile networks thanks to digital health. Additionally, it has expanded the ways in which locals can obtain health information and become aware of their health state, which has improved public health.^35^ This wave of innovation extends across countless facets of life, making healthy living not only attainable but even empowering for communities long left behind.

 By facilitating accessibility, digital health solutions aid rapid, easy, and complete access to patient records to improve health decisions. These present significant chances to raise therapy efficacy and outcomes.^36^ With emerging technologies such as new generation sequencing, digital medicines, AI and its subset, ML, and the growing need for a diverse workforce and standard of practice, the backdrop for the transformation of healthcare becomes even more apparent.^37^ Diagnostics, therapies, care delivery, regenerative treatment, and precision medicine models can all be enhanced and transformed by genomics and other technologies, such as biometrics, tissue engineering, and the vaccine sector. Disease prevention, early diagnosis, and remote chronic disease management—such as wirelessly observed therapy, which uses a cutting-edge technique to track therapy adherence—are all made possible by these technological advancements.^8,37^ In the era of disruptive and minimally invasive medicine, providing and delivering health care anywhere, whenever, is the most promising new approach. Patients and those in need of these services can access vital resources via Mobile Internet Devices (MIDs).^37,38^

 The care gaps existing among the underserved and marginalized, including pregnant women who have limited access to rural maternity, have been bridged by telehealth and other digital technologies.^39,40^ The integration of telehealth into maternal care has yielded a reformation of this subsection of the healthcare industry. Through telehealth, a drastic reduction in adverse intrapartum and postpartum outcomes, which may have been associated with delayed care initiation due to poor access to a caregiving facility, has been witnessed.^41^ Additionally, telemedicine is set to replace most in-person antenatal and postpartum supervisory care. For example, antenatal care models recommend at least 14 antenatal care contacts for low-risk pregnancies; telehealth offers a platform supplementing physical care sessions, consequently cutting down on the cost of accessing healthcare facilities and the resultant loss of work hours.^39,41-43^

 Furthermore, self-care and resultant health outcomes can be greatly optimized by remote patient monitoring. The American Congress of Obstetricians and Gynecologists advocates for and supports remote patient monitoring in the clinical management of pregnancy-associated comorbid conditions such as diabetes and gestational diabetes, hypertension in pregnancy, and preeclampsia by tracking vital signs and optimizing patients’ health parameters while cutting down on the number of required specialist visits.^44,45^ Also, teleconsultations via video conference or audio conference platforms can increase access to maternal-fetal medical care and other advanced obstetric care, which may ordinarily not be present in rural, isolated, or hard-to-reach areas. Medical collaborations between resident physicians and these specialists can expedite this process.^46,47^

 Healthcare services for advancing the management of sexually transmitted diseases can leverage digital transformation. By utilizing an array of digital tools such as web-based testing portals, self-testing models, digital health education delivery, and service promotion through mobile apps, high-quality and equitable health services can be delivered to sexual partners and key populations.^32,48^

 The digitalization of the medical industry has greatly influenced and impacted the beliefs, access to, and patronage of traditional medical services.^49^ This development impact has influenced pharmaceutical research and development, medical services-seeking behavior, and approaches to conventional medical practices.^50,51^ It now encourages the use of a mobile network to provide conventional medical services like scheduling appointments, health consultations, paying bills, and requesting medical reports.^35^ This not only helps break the mould of traditional medical services and maximizes the effectiveness of medical services, but it also optimizes the medical service process and increases the efficiency of healthcare delivery systems.^52,53^

 The pharmaceutical industry is experiencing a paradigm shift fueled by digitalization. This wave of innovation is delivering a triple punch, impacting various aspects of the development pipeline. Digital tools like big data analytics are illuminating consumer trends and unmet medical needs with unprecedented clarity, highlighting the increasing market need for pharmaceuticals. It has also facilitated the timely analysis of clinical trial data for pharmaceutical services. Consequently, it is freeing the field from the burden of high upfront costs, high risk, and drawn-out cycles.^54^

 The expenses associated with creating, sharing, and gaining access to health information are removed along with the geographical and temporal constraints through digitalization. This has been shown to improve public health by expanding the channels through which locals obtain health information.^8,35^ A shift towards healthier food choices is now being observed, a resultant effect of the abundance of dietary information across multiple digital health platforms.^55^

 Digital tools are now quite beneficial for testing and monitoring cognitive function in the elderly.^56,57^ These tools can provide objective and reliable evaluations of cognitive capacities, making it possible to make and deliver rapid geriatric healthcare policies and decisions. Digital cognitive assessments assist medical professionals in identifying subtle changes in cognitive function and subsequently enable prompt intervention with targeted and precision therapies to manage or mitigate cognitive deterioration.^58,59^

## Recommendations

 Digital health has become an effective, innovative tool that is helping break barriers, enabling rural access to health care, and advancing early detection of infectious diseases. However, there are still impediments to its productivity, acceptability, and accessibility. Barriers to digital healthcare acceptability can be addressed by creating and implementing targeted health policies at the different levels of healthcare delivery. These policies must reflect the sociocultural norms and practices of those accessing healthcare with these tools. Considering digital divides, economic standards, and literacy levels, it is also pertinent to put structures in place that will facilitate access to or ownership of these devices among inhabitants of the lower economic strata. Affordable internet access, bridging infrastructural gaps, and digital literacy programmes are a few of these measures. Human resource development in terms of availability of manpower, capacity building, and better remuneration are other means by which healthcare access can be improved in Africa. It is recommended that a system of monitoring and evaluation be highly prioritized as this would improve the poor maintenance culture and poor implementation puzzles limiting Africa’s growth.^60^ Technological companies, tech leaders, and developmental organisations should incorporate technological solutions addressing the needs of people with disabilities and marginalized communities. By investing in the research and development of assistive technologies and user interfaces that empower people with disabilities and bridge the digital divide, they can contribute their quota to achieving fairness and equitable healthcare access.

## Conclusion

 The rapid proliferation of information, communication, and technology is transforming healthcare delivery across the globe. The narrative holds true even in the African context, although various factors have retarded the pace of change in the dynamics and rhythm of healthcare delivery in Africa. With increasing diversity among the world’s population in terms of socio-cultural background, religious beliefs, and moral philosophies, there is an increasing need to vary the approaches to healthcare delivery to meet the healthcare needs of various populations, including key populations, and achieve health equity. Digital health interventions promise a platform not only to transform but also to ensure universal health coverage. Bridging the healthcare gaps by addressing major challenges in the African healthcare delivery system, such as limited resources, poor administration, political instability, and inadequate infrastructure, promises a major leap. Leveraging digital health, such as remote healthcare monitoring, telehealth, smart devices, the healthcare internet of things (IoT), and AI, can significantly attenuate the burden faced by the African population in accessing health care. A ‘one-size-fits-all’ approach is unsuitable in the African context, considering her unique diversity. This calls for the enactment of health policies that advocate tailored approaches to healthcare delivery. Investing in context-specific health interventions that address the specific challenges and priorities of different communities is sacrosanct. The ultimate goal is to create a more efficient and effective healthcare system that meets the needs of the African population.

## Acknowledgements

 We appreciate Global Health Focus for constantly raising global researchers all over Africa.

## Competing Interests

 The authors declare that they have no competing interests.

## Ethical Approval

 This study used data freely accessible and downloadable in the public domain. Therefore, institutional review board approval was not required.
